# Talin1 knockdown prohibits the proliferation and migration of colorectal cancer cells via the EMT signaling pathway

**DOI:** 10.3892/ol.2021.12943

**Published:** 2021-07-27

**Authors:** Ling Ji, Feizhao Jiang, Xianping Cui, Chengkun Qin

Oncol Lett 18: 5408-5416, 2019; DOI: 10.3892/ol.2019.10902

Following the publication of this paper, the authors contacted the office to inform us that they had made errors in the compilation of the tumour images shown in Fig. 4A. Further to the contact we received from these authors, an independent enquiry was conducted in the Editorial office, after which we were able to ascertain that the data in question had appeared in slightly modified form in another publication written by different authors 6 years previously. Owing to the fact that the contentious data in the above article had already been published elsewhere prior to its submission to *Oncology Letters*, thereby undermining our confidence in the presented data, the Editor has decided that this paper should be retracted from the Journal. The authors were asked for an explanation to account for these concerns, but the Editorial Office did not receive a satisfactory response. The Editor apologizes to the readership for any inconvenience caused.

